# Legislation Related to E-Cigarettes in The Gulf Cooperation Council Countries

**DOI:** 10.31557/APJCP.2020.21.12.3449

**Published:** 2020-12

**Authors:** Mohamad Chehab

**Affiliations:** *Hamad Medical Corporation, Qatar. *

**Keywords:** Legislation, e-cigarettes, GCC

## Abstract

Even though 90% of the world’s population is covered by the World Health Organization’s Framework Convention on Tobacco Control, it is estimated that only one-fifth (21%) and half (49%) of the countries that ratified this convention will achieve the target of reducing tobacco use by 30 % among men and women respectively in 2025. Over the past decade, there has been an increasing trend in the use of electronic cigarettes or e-cigarettes for recreational use as well as smoking cessation. In concurrence with the increasing use of e-cigarettes among smokers of different age groups, nations have developed relevant national regulations on its sale, advertisement, packaging, product regulation, taxation, and surveillance. The countries of the Gulf Cooperation Council (GCC) region have also witnessed several legislations, at varying extent, related to the use of electronic cigarettes. However, the evidence on the use of electronic nicotine delivery systems remains scarce in the GCC region. Thus, further research on this emerging public health issue is warranted to generate the evidence necessary for the formulation of comprehensive tobacco control laws and effective prevention strategies.

## Introduction

Despite the fact that 90% of the world’s population is covered by the World Health Organization’s Framework Convention on Tobacco Control (WHO, 2015), it is estimated that only one-fifth (21%) and half (49%) of the countries that ratified this convention will achieve the target of reducing tobacco use by 30 % among men and women respectively in 2025 (Bilano et al., 2015).

During the past decade, there has been an increasing trend in the use of electronic cigarettes or e-cigarettes for recreational use as well as smoking cessation. However, its use for the latter purpose has been an area of controversy due to the lack of stringent evidence on the efficacy and safety of such method (Mcgraw, 2014). In concurrence with the increasing use of e-cigarettes among smokers of different age groups, 98 nations have developed relevant national regulations on the sale, advertisement, packaging, product regulation, taxation, and surveillance (WHO, 2014). Regarding electronic cigarettes or e-cigarettes, the literature shows that 68 nations currently have regulations for these devices. Moreover, there are different types of regulations in effect across the aforementioned countries such as existing regulations, new policies specific to e-cigarettes, and amendments of existing legislation (Kennedy et al., 2016).

*Regional Situation in the GCC*

The Gulf Cooperation Council (GCC) is comprised of six countries: Qatar, Bahrain, Oman, Kuwait, Saudi Arabia, and United Arab Emirates (Khoja et al., 2017). The total population in the region has increased from 44.3 million up to 53.5 million between 2010 and 2016, with less than two-thirds (59.4%) living in Saudi Arabia. The population is expected to further grow and reach 106.8 million by 2,033. Also, the proportion of expatriates in the GCC countries, excluding the United Arab Emirates, was 40.8% in 2016 (GCC-Stat, 2019). 

The regional countries have unanimously ratified the World Health Organization’s Framework Convention on Tobacco Control between 2004 and 2007, with Qatar being the first to ratify on 23^rd^ July 2004 and Bahrain being the last on 20^th^ March 2007 (WHO, 2017). However, tobacco control in the region dates back to the sixth Health Ministers’ Conference in 1979 when the Kingdom of Saudi Arabia submitted a working paper on the aforementioned issue (GHC, n.d.).

*Bahrain*

The total population in the Kingdom of Bahrain was estimated at 1,423,726, with expatriates accounting for 53.3% (GCC-Stat, 2019). The first tobacco control law (no.10) was issued in 1994 by the country’s authorities and regulated the growth, consumption, sponsorship, and sales of tobacco. The decree also paved the way for the formation of the National Tobacco Control Committee in 1995 (Fadhil, 2007). The aforementioned law was followed by a series of decrees, resolutions, and decisions. In 2013, decision no.38 was enacted and banned the importation, distribution, and selling of e-cigarettes in the kingdom (Legalaffairs.gov.bh, 2013). 

*Kuwait*

It is estimated that 4,082,704 people lived in the State of Kuwait populous during 2016, almost a third (31.1%) of who were citizens (GCC-Stat, 2019). The first tobacco control law (no.15) was issued in 1995 and regulated the growth, consumption, sponsorship, and sales of tobacco. Law no.15 was followed by law no.42 in 2014, decision no.916 in 2015, decision no.288 in 2016, and decision no.6 in 2016. Kuwait is the only GCC country where the legal age for purchasing any tobacco product is 21 years. Decision no.6 included electronic cigarettes, shishas, and other devices as part of the regulations imposed on tobacco products in the country (Emro.who.int, n.d.). Thus, e-cigarettes are not prohibited in the State of Kuwait. 

*Oman*

4,414,051 people inhabited the Sultanate of Oman in 2016, with nationals accounting for 55% of the population (GCC-Stat, 2019). Ministerial decree no.55 was one of the first to ban smoking, specifically in the premises of the Ministry of Health. The decree superseded by several decrees, decisions, and legislations up to ministerial decree no.43 in 2018 that banned the advertisement of tobacco products in the sultanate. In 2015, ministerial decree no.698 banned the use of e-cigarettes and shishas and ordered the immediate market withdrawal of any products in the country (Emro.who.int, n.d.).

*Qatar*

The State of Qatar was home to 2,617,634 nationals and expatriates in 2016 (GCC-Stat, 2019). Law no.20 of 2002 was one of the first tobacco-specific laws in the country and consisted of 17 articles related to the control of tobacco and its derivatives. The law was succeeded by a series of circulars, laws, and decrees. The most recent circular was in 2019 regarding the warning labels on the packages of cigarettes. The most recent law was law no.10 in 2016 that consisted of 25 articles, including article 7 that prohibits electronic cigarettes and shishas in the country (Emro.who.int, n.d.).

*Saudi Arabia*

In 2016, the population of the Kingdom of Saudi Arabia reached 31,787,580 with more than a third (36.8%) being expatriates (GCC-Stat, 2019). The earliest tobacco-related legislation in the country was a circular in 1983 that prohibited smoking in ministries and governmental institutions. The circular was followed by a series of royal and ministerial decrees, including a commissioner approval (No.46) on the formation of a national tobacco control committee in 2007 as well as an anti-smoking law (royal decree No.M/56) in 2015. Similarly, a ministerial decision in 2015 prohibited the importation, selling, and distribution of electronic cigarettes, shishas, and similar devices in alignment with the decisions of the 75th Conference of GCC Health Ministers’ Council (Emro.who.int, n.d.).

*United Arab Emirates (UAE)*

The UAE was inhabited by 9,121,167 citizens and expatriates in 2016 (GCC-Stat, 2019). One of the first anti-tobacco legislations was the ministerial circulation no.1 in 1996. The circulation banned smoking in the premises of the Ministry of Health and all governmental health institutions. Subsequently, a series of ministerial decrees and circulations followed as well as the federal tobacco control law no.15 in 2009. As a result, the importation and selling of e-cigarettes and e-shishas was prohibited in the country. However, the UAE is the sole GCC country that allows the growth and manufacturing of tobacco on its soil based on article 15 of ministerial decision no.24 of 2013 (Emro.who.int, n.d.).

In conclusion, during the review of literature, it was found that evidence is scarce on the use of electronic nicotine delivery systems in the GCC region. Thus, further research on this emerging public health issue is warranted to generate the evidence necessary for the formulation of comprehensive tobacco control laws and effective prevention strategies.
